# Implementing traumatic brain injury screening in behavioral health treatment settings: results of an explanatory sequential mixed-methods investigation

**DOI:** 10.1186/s13012-023-01289-w

**Published:** 2023-08-16

**Authors:** Kathryn A. Hyzak, Alicia C. Bunger, Jennifer Bogner, Alan K. Davis, John D. Corrigan

**Affiliations:** 1 Department of Physical Medicine and Rehabilitation, The Ohio State University College of Medicine, Columbus, OH 43210-1234 USA; 2 College of Social Work, The Ohio State University, Columbus, OH USA; 3 Department of Psychiatry and Behavioral Sciences, Center for Psychedelic and Consciousness Research, Johns Hopkins University Baltimore, Baltimore, MD USA; 4 Department of Psychiatry, College of Medicine, The Ohio State University, Columbus, OH USA; 5 Ohio Valley Center for Brain Injury Prevention and Rehabilitation, The Ohio State University Wexner Medical Center, Columbus, OH USA

**Keywords:** Mixed methods, Traumatic brain injury, OSU TBI-ID, Behavioral health, Theory of Planned Behavior, Structural equation modeling, Implementation science

## Abstract

**Background:**

Traumatic brain injury (TBI) is a complex condition common among individuals treated in behavioral healthcare, but TBI screening has not been adopted in these settings which can affect optimal clinical decision-making. Integrating evidence-based practices that address complex health comorbidities into behavioral healthcare settings remains understudied in implementation science, limited by few studies using theory-driven hypotheses to disentangle relationships between proximal and medial indicators on distal implementation outcomes. Grounded in the Theory of Planned Behavior, we examined providers’ attitudes, perceived behavioral control (PBC), subjective norms, and intentions to adopt The Ohio State University TBI Identification Method (OSU TBI-ID) in behavioral healthcare settings.

**Methods:**

We used an explanatory sequential mixed-methods design. In Phase I, 215 providers from 25 organizations in the USA completed training introducing the OSU TBI-ID, followed by a survey assessing attitudes, PBC, norms, and intentions to screen for TBI. After 1 month, providers completed another survey assessing the number of TBI screens conducted. Data were analyzed using structural equation modeling (SEM) with logistic regressions. In Phase II, 20 providers were purposively selected for semi-structured interviews to expand on SEM results. Qualitative data were analyzed using thematic analysis, integrated with quantitative results, and combined into joint displays.

**Results:**

Only 25% (55/215) of providers adopted TBI screening, which was driven by motivations to trial the intervention. Providers who reported more favorable attitudes (*OR*: 0.67, *p* < .001) and greater subjective norms (*OR*: 0.12, *p* < .001) toward TBI screening demonstrated increased odds of intention to screen, which resulted in greater TBI screening adoption (*OR*: 0.30; *p* < .01). PBC did not affect intentions or adoption. Providers explained that although TBI screening can improve diagnostic and clinical decision-making, they discussed that additional training, leadership engagement, and state-level mandates are needed to increase the widespread, systematic uptake of TBI screening.

**Conclusions:**

This study advances implementation science by using theory-driven hypothesis testing to disentangle proximal and medial indicators at the provider level on TBI screening adoption. Our mixed-methods approach added in-depth contextualization and illuminated additional multilevel determinants affecting intervention adoption, which guides a more precise selection of implementation strategies.

**Supplementary Information:**

The online version contains supplementary material available at 10.1186/s13012-023-01289-w.

Contributions to the literature

- We utilized theory-driven hypothesis testing to disentangle relationships between proximal (attitudes, norms, and perceived behavioral control) and medial (intentions) indicators to adoption of The Ohio State University TBI Identification Method in behavioral healthcare settings.
- One-quarter of providers adopted TBI screening, which was driven by favorable attitudes, greater subjective norms, and greater intentions to screen for TBI.
- Although providers reported that TBI screening can improve diagnostic and clinical decision-making, additional training, leadership engagement, and state-level mandates are needed to increase the widespread, systematic uptake of TBI screening.
- Implementation strategies targeting provider-level, inner-setting, and outer-setting determinants can be selected with greater precision.

## Background

Complex physical health comorbidities are common among individuals with substance use and mental health conditions [1]; however, less attention has been directed toward identifying and addressing these comorbidities through evidenced-based practice (EBP) integration in behavioral healthcare settings. Traumatic brain injury (TBI) is an example of a common, yet under-identified chronic condition among individuals who seek treatment for substance use or mental health problems in behavioral healthcare settings [2]. An estimated 60% of individuals in these settings have a lifetime exposure to TBI that affects their ability to fully engage in and benefit from treatment [3, 4]. Implementing universal screening for lifetime exposure to TBI is critical to preventing misdiagnoses and/or mislabeling clients as poorly motivated or “non-compliant” with treatment due to chronic cognitive and behavioral problems resulting from TBI [4].

The Ohio State University TBI Identification Method (OSU TBI-ID) is one of the most established screening methods for evaluating lifetime exposure to TBI across various populations [5–8]. This screening method was first validated among clients seeking treatment for substance use disorders in behavioral health treatment settings [9, 10] and can be completed in 3–5 min. Reliability of the OSU TBI-ID has been demonstrated by both inter-rater and test/re-test reliability [9–12]. Initial validation studies showed that OSU TBI-ID indices of lifetime TBI exposure predicted current affective, behavioral, or cognitive deficits [9, 10]. Additional studies demonstrated correspondence between contemporaneous medical records in childhood and adult self-report [13] and an association between OSU TBI-ID findings, and abnormalities were observed via magnetic resonance imaging, functional magnetic resonance imaging, diffusion tensor imaging, and proteomics [14–20].

The OSU TBI-ID uses gold standard interview techniques to prompt an individual’s memory about possible injuries to the neck and head to determine what injuries resulted in a TBI, including injury severity (based on length of altered state or loss of consciousness), most recent injury, age at first injury, and multiple repeated injuries (i.e., repetitive blows to the head due to domestic violence). Formal diagnostic procedures like neuroimaging or neuropsychological assessment are not only time-consuming, but they are also not sensitive to an individual’s lifetime exposure to TBI. Single questions such as “Have you ever sustained a TBI?” are likely to under-identify exposure in clients who may not know they have sustained a TBI (including concussions) because they never sought treatment or their TBI was not identified during treatment. Screening for TBI in behavioral healthcare settings is the first step toward individualizing and optimizing behavioral health treatment and interventions. Like many other EBPs, the OSU TBI-ID remains underutilized in behavioral healthcare settings which potentially limits the quality of care provided for a substantial number of individuals with comorbid TBI and behavioral health conditions. Numerous multilevel determinants can affect the uptake of EBPs at different stages in the implementation life cycle, particularly in treatment environments like behavioral healthcare. At the early phases of implementation, research and theory consistently point to characteristics of providers as primary determinants (i.e., barriers and facilitators) and/or mechanisms to EBP adoption [21–28]. The Theory of Planned Behavior (TPB) posits that provider attitudes (the degree to which a person has a favorable or unfavorable opinion about the target behavior), perceived behavioral control (PBC; the degree to which the individual believes they can perform the behavior), and subjective norms (pressures to perform the behavior) directly affect one’s intentions to perform that behavior and, ultimately, behavior performance [28]. In other words, providers who have more favorable attitudes toward screening for TBI, higher perceived control over TBI screening, and greater social pressures to screen for TBI will have greater intentions to screen and, ultimately, will be more likely to screen for TBI. The TPB has been widely used in other implementation science studies [26, 27, 29–31] to guide specification of relationships between proximal indicators (i.e., attitudes, PBC, subjective norms) and mediators (i.e., intentions) on distal implementation outcomes (i.e., EBP use) to help clarify where early-phase implementation succeeds or fails [32]. However, the extent to which each of these constructs affects EBP adoption is not universal, leading us to investigate the extent to which these provider-level characteristics affect TBI screening adoption so that implementation strategies can be more precisely selected and tailored to steer EBP uptake.

Yet, even when individual-level characteristics are studied as potential determinants to EBP adoption, they are rarely contextualized within the broader service setting, leaving questions about *why* these determinants did or did not affect adoption. Qualitative insights from providers can improve our understanding about their attitudes or social pressures, for example, to conduct TBI screening, as well as illuminate additional determinants that may be affecting adoption. Therefore, this study extends the current literature on TPB applied to TBI screening adoption in behavioral health care using mixed methods.

We present the first sequence of results from our published protocol aimed to investigate the adoption of the OSU TBI-ID in behavioral healthcare settings [32]. Specifically, the first aim of this study was to examine the relationships between behavioral health providers’ attitudes, PBC, and subjective norms as predictors to TBI screening intentions and to examine whether intentions to adopt TBI screening mediate TBI screening behaviors at a 1-month follow-up. We hypothesized that providers who had more favorable attitudes, greater PBC, and greater perceived social norms to screen for TBI would demonstrate greater intentions to screen for TBI and, subsequently, greater odds of adopting TBI screening at the 1-month follow-up. Our second aim was to expand upon and contextualize the quantitative results using semi-structured interviews with a subset of behavioral health providers to gain deeper insights into factors affecting TBI screening adoption.

## Methods

### Study design

This was an explanatory sequential mixed-methods study (QUANT → qual) [33, 34]. Details about this mixed-methods design and rationale are published elsewhere [32]. We use the Journal Article Reporting Standards for Mixed Methods Research for transparency of reporting our research (Supplemental file 1) [35]. This study was approved by the Institutional Review Board at The Ohio State University (OSU).

### Participants and setting

Participants included 215 licensed behavioral health providers (e.g., licensed psychologists, social workers, professional clinical counselors) employed in behavioral health treatment settings throughout the USA (e.g., community-based substance use treatment and/or mental health clinics, hospital-based outpatient clinics, domestic violence organizations).

### Phase I

#### Recruitment and data collection

To enhance representativeness of our sample, we recruited providers through multiple sources, including the Star Behavioral Health Providers Program (SBHP) of Ohio (Sample 1), Google searches and personal referrals (Sample 2), a continuing education listserv at OSU (Sample 3), and the National Association for Alcoholism and Drug Abuse Counselors (Sample 4). Participants were recruited between November 2020 and January 2022.

At Time 1, providers were emailed a detailed study description, study inclusion criteria, informed consent, and a Qualtrics survey link that included a 45-min PowerPoint module on the OSU TBI-ID. The module consists of the following: (1) an introduction to and importance of using the OSU TBI-ID to screen for TBI in behavioral healthcare settings, (2) a downloadable PDF version of the OSU TBI-ID screening form, (3) video-based case exemplars demonstrating how to administer the OSU TBI-ID with clients, and (4) how to interpret the findings. This training module was created in 2014 via a collaboration between the authors and WETA-TV, the Public Broadcasting Service station in Washington DC. The OSU TBI-ID forms and training are available free online for any professional who wishes to become trained in administering the screening method and hence was selected for use in this study. Completion of the module was followed immediately by a survey assessing providers’ attitudes, PBC, subjective norms, and intentions to use the OSU TBI-ID with clients over the next month. Notably, providers from Sample 1 (*n* = 15) already completed a similar in-person TBI education program through SBHP, and hence did not receive the web-based module. Providers received one continuing education credit for completing the training and were entered into a raffle for the chance to win a US $50 gift card; 60 winners were selected at random.

At Time 2, providers were sent a second email 1-month following completion of the first survey that included a Qualtrics link asking the self-reported number of TBI screens conducted with clients over the previous month. The Dillman method [36] was applied to increase the response rate between the two timepoints. Specifically, participants received up to six total contacts, where a follow-up email was sent approximately 1 week after initial contact, two additional emails were sent at 4 and 7 weeks, followed by two biweekly emails. The response rate between Times 1 and 2 was 74.4%. Surveys were linked using providers’ first and last names, email address, and a unique digital identifier which they provided at the end of both surveys. Providers were entered into a raffle for the chance to win a US $25 gift card for completing the second survey; 20 winners were selected at random.

#### Main constructs and measures

Constructs, their definitions, and timing of measurement are in Table 1. The 28-item Theory of Planned Behavior Questionnaire for TBI (TPBQ-TBI) was used to measure provider attitudes, subjective norms, PBC, and intentions at Time 1 and TBI screening behaviors at Time 2. We defined adoption according to the Proctor et al. (2011) definition and operationalized it as providers’ intentions to screen for TBI and providers’ utilization of the OSU TBI-ID measured by the number of TBI screens conducted. The TPBQ-TBI was adapted based on previously published TPBQ measures, where items were tailored to reference the OSU TBI-ID used for this study [37, 38]. Twenty-four items were retained [37], and four were adapted [38] from the published measures. Items on the attitudes, PBC, norms, and intentions subscales were measured on a 7-point Likert scale from 1 (*strongly disagree*) to 7 (*strongly agree*) and averaged for a total score. Higher scores reflected more favorable attitudes, greater PBC, stronger norms, and greater intentions associated with using the OSU TBI-ID. Each subscale demonstrated high internal consistency reliability (*α* = 0.77–0.94) [37]. TBI screening behaviors were measured at Time 2 through provider self-report.

**Table 1:** Key constructs, definitions of constructs, and timing of measurement

Construct	Definition	Measure	Timepoint	Variable
Attitudes	“The degree to which a person has a favorable or unfavorable evaluation or appraisal of the behavior in question” [16]	TPBQ-TBI	1	Predictor
Perceived behavioral control	“Perception of the ease or difficulty of performing the behavior of interest” [16]	TPBQ-TBI	1	Predictor
Subjective norms	“The perceived social pressure to perform or not to perform the behavior” [16]	TPBQ-TBI	1	Predictor
Adoption	“*Intention*, initial decision, or *action* to try or employ an innovation or evidenced-based practice” [3] and is sometimes referred to as “uptake” of an EBP	TPBQ-TBI	1,2	Intention (mediator) Number of TBI screens conducted (primary outcome)

This table is adapted from Coxe-Hyzak, K. A., Bunger, A. C., Bogner, J., Davis, A. K., Corrigan, J. D. Implementing traumatic brain injury screening in behavioral healthcare: protocol for a prospective mixed-methods study. *Implement Sci Commun*. 2022 Dec;3(1):17

### Statistical analyses

We used SPSS v.27 to analyze descriptive data [39]. We compared data for each subsample using Pearson chi-square tests for categorical variables or Fisher’s exact tests. We compared continuous variables using one-way ANOVA and post hoc tests using Tukey–Kramer comparisons to account for unequal sample sizes among the four samples [40]. Response options from each subscale were collapsed into six categories due to low cell counts on the lower scores. In addition, due to the right-skewed nature of the TBI screening data, we recoded counts as binary (1 = yes, screened for TBI; 0 = no, did not screen for TBI). We used descriptive statistics to determine differences between demographic variables on the main study outcome (i.e., TBI screening behaviors) (see Table 2). We also assessed for differences between the two timepoints and found some statistically significant differences on the number of licensed professional counselors who participated in both surveys (*p* = 0.03), as well as differences between providers employed in private practice settings, hospital-based inpatient settings, and managed care organizations (*p* = 0.01). Finally, because providers in the SBHP sample received a different educational program than the other three samples, we conducted a sensitivity analysis to determine if excluding this sample affected the main outcome; however, no differences were detected (*p* > .05), and therefore, this sample was retained. However, we used this sample as a control for advanced analyses due to differences on key constructs of the TPB. See Supplemental files 2 and 3 for differences between Phase I subsamples.

**Table 2:** Differences between demographic characteristics on TBI screening behaviors

	Screened 55 (25.6%)	Did not screen 160 (74.4%)	*p*-value^a^	Effect size
Sample set			0.05	0.04b
Sample 1	4 (7.3)	11 (6.9)		
Sample 2	9 (16.4)	14 (8.8)		
Sample 3	25 (45.5)	105 (65.6)		
Sample 4	17 (30.9)	30 (18.8)		
Age group			0.99	0.00b
18–24	1 (1.8)	3 (1.9)		
25–34	11 (20.0)	43 (26.9)		
35–54	25 (45.5)	72 (45.0)		
55–65	12 (21.8)	37 (23.2)		
> 65	5 (9.1)	12 (7.5)		
Gender				
Female	47 (85.5)	134 (84.5)	0.81	0.00b
Male	7 (12.7)	23 (14.4)		
Nonbinary	0 (0.0)	1 (0.1)		
Race/ethnicity			0.92	0.01b
Caucasian or White	44 (80.0)	132 (82.5)		
African American or Black	5 (9.1)	11 (6.9)		
Multi-racial	2 (3.6)	9 (5.6)		
Hispanic or Latinx	2 (3.6)	4 (2.5)		
Asian or Pacific Islander	1 (1.8)	1 (0.1)		
Other	1 (1.8)	3 (0.02)		
Highest level of education			0.02*	.16^b^
Masters or doctorate	49 (89.1)**	117 (73.1)**		
Associates or bachelors	6 (10.9)**	40 (25.0)**		
Behavioral health setting			0.03*	0.33^b^
Private practice	23 (41.8)**	34 (21.3)**		
Community-based outpatient clinic	13 (23.6)	42 (26.3)		
Hospital-based outpatient services	9 (16.4)	16 (10.0)		
Residential treatment facility	4 (7.3)	4 (2.5)		
Prison/jail	2 (3.6)	10 (6.3)		
School-based behavioral health	1 (1.8)	10 (6.3)		
Senior services	1 (1.8)	4 (2.5)		
Hospital-based inpatient services	0 (0.0)	9 (5.6)		
Managed care organization	0 (0.0)	4 (2.5)		
Public health agency	0 (0.0)	3 (1.9)		
Child welfare agency	0 (0.0)	9 (5.6)		
Other^c^	2 (3.6)	11(6.9)		
Years worked as a behavioral health provider (M, SD)	14.2 (10.4)	13.9 (9.6)	0.85	0.03b
Years worked at the current organization (M, SD)	5.67 (6.3)	7.6 (7.9)	0.11	0.25b

^a^Based on chi-square, one-way ANOVA, or independent samples *t*-test

^b^Small effect. Note: Effect sizes are based on eta-squared, Phi, or Cohen’s D and interpreted using standard cutoffs for the respective statistical test

^c^Other behavioral health settings include primary care, military-based treatment setting, homeless shelter, community outreach and crisis center, affordable housing agency, employee assistance program, domestic violence shelter, local government authority, university academic medical institute, and professional ice hockey organization. In this group, only one provider from a domestic violence agency and one provider from the professional ice hockey organization screened for TBI

^*^Significant at the *p* < .05 level

^**^Post hoc analyses demonstrated significant differences at the *p* < .05 level

#### Structural equation model

Fit of the measurement model was determined prior to testing the general structural model [41]. A nonsignificant *χ*^2^ value was sought, but not required [42, 43]. We used the following fit indices and cutoffs: Comparative Fit Index (CFI, > 0.95), Tucker-Lewis index (TLI, > 0.95), standardized root-mean-square residual (SRMR, < 0.80), and the point estimate and 90% CI of the RMSEA (< .06) [42]. Two of the indicators on “Intent” were highly correlated with each other (*r* = 0.987), and therefore, these three indicators were computed as a mean value, and “Intent” was measured as an observed variable and excluded from the measurement model [44].

Next, structural equation modeling (SEM) with logistic regressions was conducted in Mplus 8.5 [45]. In the model for this study, “Attitudes,” “PBC,” and “Subjective Norms” were the exogenous variables hypothesized to have direct effects on the endogenous variable, “Intent,” and an indirect effect through “Intent” on the endogenous variable, “TBI screening behavior.” In addition, ‘Attitudes,’ ‘PBC,’ and ‘Subjective Norms’ were also tested for possible direct effects on TBI screening behaviors. Next, to control for sample differences, Sample 1 was included as a covariate. Because the TPBQ-TBI items are measured using ordinal response options, the robust weighted least-squares mean and variance (WLSMV) estimator was used [46].

#### Power calculation

Using standard power and RMSEA specifications for determining sample sizes in SEM and the sample size computation in R, 53 participants were needed to sufficiently power the model with an alpha level of *p* < .05, *df* = 408, power level of 0.80, and RMSEA_alternative_ = .06 [47, 48]. The final sample for this study was *N* = 215, which exceeded the minimum requirements and standard conventions for sample sizes in SEM [44].

A missing values analysis (MVA) was conducted using Little’s missing completely at random (MCAR) test in SPSS to determine percentage and patterns of missing data [49]. The MCAR test was not statistically significant (*χ*^2^ = 301.69, *df* = 282, *p* = 0.20) and missing data were less than 2% on variables with any missing data, which is not likely statistically or clinically significant [50]. Mplus uses full information maximum likelihood (FIML) for handling any missing data on the indicator variables of latent factors, as well as observed variables and covariates pulled into the model [45]. Among the 215 cases in this analysis, the minimum covariance coverage value of 0.100 was met [51].

### Phase II

#### Data collection

Twenty providers who completed Phase I surveys were purposively selected using nonrandom, maximum variation sampling [52]. Sample size was determined a priori based on a phenomenological research approach [53]. Consistent with this explanatory sequential mixed-methods design [54, 55], participants were first selected based on their individual TBI screening behaviors to capture greater detail regarding *why* TBI screens were or were not conducted within their treatment setting. Specifically, we aimed to recruit providers with a broad range of screening behaviors so that we could better understand determinants affecting decisions for or against screening adoption. In addition, since most providers from Phase I were employed in private practices, providers employed in these settings took priority over other practice settings. However, to ensure sample variation in capturing differences in contextual determinants perceived to affect TBI screening adoption, providers from a variety of behavioral health settings and states were also selected. Ongoing assessment of the sample throughout the data collection process was conducted to confirm that participants and their responses corresponded to the quantitative survey data [56].

Providers were contacted directly by email using the emails provided in Phase I. All interviews were conducted through Zoom videoconferencing software and audio-recorded with the participants’ consent. Interviews lasted approximately 35 min. Participants received a US $30 gift card for participation.

#### Qualitative interview guide

A semi-structured interview guide was developed using results from Phase I [57]. Interview questions were structured according to each of the main study constructs to ensure linkage between the two phases [54, 58]. The interview guide aimed to corroborate and expand understanding of how the provider-level characteristics affected TBI screening adoption within the treatment context [58]. See Table 3 demonstrating how the quantitative and qualitative questions were matched based on key constructs. The interview guide included nine open-ended primary and seven probing questions linked back to the main constructs from the TPB [58].

**Table 3:** Examples of matched quantitative and qualitative questions situated by theoretical construct

Quantitative questions	Qualitative questions
*Attitudes*	
• Screening for TBI using the OSU TBI-ID fits with my practice preferences	Regardless of whether or not you used the OSU TBI-ID in your work, what are your thoughts about screening for TBI using the OSU TBI-ID in your practice?
• Using the OSU TBI-ID to screen for TBI will result in improved outcomes for my clients	
*Subjective norms*	
• Those whose opinions I value would prefer that I screen for TBI using the OSUTBI-ID with my clients	What are the expectations in your practice setting or organization about implementing new interventions?
• My colleagues think I should use the OSU TBI-ID to screen for TBI with my clients	
*Perceived behavioral control*	
• I am confident that I could screen for TBI using the OSU TBI-ID with new and/or established clients over the next month	How easy or difficult was it to use the OSU TBI-ID to screen for TBI with your clients? Please explain
• I have access to the resources and opportunities I need to use the OSU TBI-ID	
*Intentions*	
• It is likely that I will use the OSU TBI-ID to screen for TBI in my practice with clients over the next month • Chances are that I will use the OSU TBI-ID in my practice with clients over the next month	When you were first introduced to this TBI screening method, what were your plans to try to use this screening method with your clients to screen for TBI? Please explain
*TBI screening behaviors*	
• How many new clients did you screen for TBI using the OSU TBI-ID over the last month? • How many returning clients did you screen for TBI using the OSU TBI-ID over the last month?	[If participant *did not* screen for TBI] What were some of the reasons why you did not use the OSU TBI-ID with your clients? [If participant *did* screen for TBI] What facilitated your use of the OSU TBI-ID in your work?

#### Qualitative data analysis

Interviews were transcribed verbatim immediately upon interview completion and then cleaned and prepared for data analysis [58]. All interview data were managed and analyzed using NVivo 12.0 [59]. Next, codes were generated deductively according to the five main constructs from the TPB [60]. The data were coded into these primary categories to allow for initial organization of the qualitative data, to frame the analysis according to the primary study purpose, and to prepare the qualitative data to be mixed with the quantitative data during the mixed-methods analysis stage [58, 61].

Next, two coders independently familiarized themselves with the data by reading each transcript, taking notes, and creating additional codes within each main construct. Using an iterative process, the two coders met to discuss the initial set of codes and to discuss similarities and differences on each set of codes [58]. Coders then returned to the data to refine codes into main, overarching themes [60, 62]. Supportive quotes were selected to represent the essence of each theme and provide context to the themes [60].

### Mixed-methods data integration and analysis

**Fig. 1:**
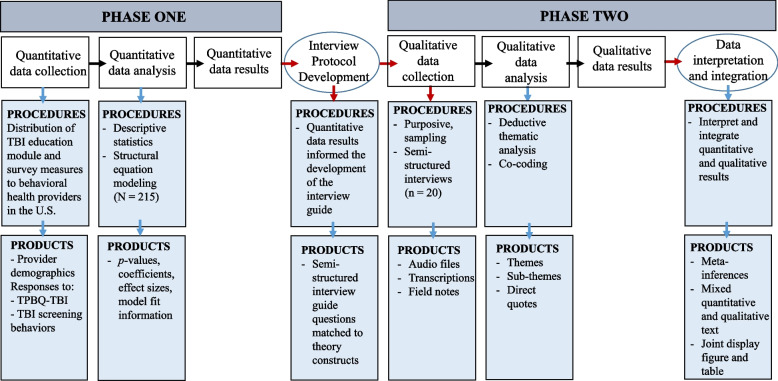
Procedural diagram for the explanatory sequential mixed-methods design

Several points of data integration were used [54, 58, 63]. First, results from Phase I were used to guide the selection of participants to recruit for Phase II qualitative interviews [58]. Second, results from Phase I to Phase II were connected by using the quantitative results to develop the qualitative interview guide [64]. Third, results from both phases were mixed through meta-inferences drawn from assessing the combination of quantitative and qualitative data [58]. During this stage, data were merged by examining both sets of data side by side to assess for confirmation, expansion, or discordance. Confirmation occurred when the quantitative and the qualitative results lead to the same conclusion and the data from each reinforced the other [58]. Expansion occurred when the quantitative and qualitative results had the same commonalities and conclusions, but additional, nonoverlapping interpretations were made when qualitative data further explained the quantitative results [58]. Discordance occurred when the quantitative and qualitative results did not match, leading to conflicting interpretations [58]. Fourth, data integration occurred through weaving, where the quantitative and qualitative results are presented within the text side by side [54, 57, 58, 61]. Finally, joint displays were created for visual depictions of the mixed results, presenting both quantitative and qualitative data together [54, 58, 61]. Red arrows in Fig. 1 represent points of data integration throughout the study.

## Results

Of the 215 participants who completed surveys, most identified as female (85.4%), Caucasian or White (81.9%), and reported earning a masters or doctoral degree (78.3%). Most participants were licensed social workers (*n* = 128) or counselors (*n* = 43). About one-quarter of participants reported being employed in private practice settings (26.5%, *n* = 57) or in community-based outpatient treatment clinics (25.6%, *n* = 55). Overall, participants were employed in their current organization for about 7 years (*SD* = 7.57). Additional sample characteristics are provided in Table 4.

**Table 4:** Characteristics of participants in the quantitative and qualitative phases

	Quantitative phase *N* = 215	Qualitative phase *N* = 20
	*n* (%)	*n* (%)
Age group		
18–24	4 (1.9)	0
25–34	45 (21.2)	1 (5.0)
35–54	97 (45.8)	13 (65)
55–65	49 (23.1)	6 (30.0)
> 65	17 (8.0)	0
Gender		
Female	181 (85.4)	18 (90.0)
Male	30 (14.2)	2 (10.0)
Nonbinary	1 (0.5)	0
Race/ethnicity		
Caucasian or White	176 (81.9)	19 (95.0)
African American or Black	16 (7.4)	1 (5.0)
Multi-racial	11 (5.1)	0
Hispanic or Latinx	6 (2.8)	0
Asian or Pacific Islander	2 (0.9)	0
Other^a^	4 (1.8)	0
Highest level of education		
Masters or doctorate	166 (78.3)	17 (85.0)
Associates or bachelors	46 (21.7)	3 (15.0)
License type		
LSW	58 (27.0)	3 (15.0)
LISW-S	47 (21.9)	5 (25.0)
LPC	24 (11.2)	4 (20.0)
LISW or LCSW	23 (10.7)	1 (5.0)
LICDC	22 (10.2)	3 (15.0)
LPCC or LPCC-S	20 (9.3)	5 (25.0)
LCDC-II or LCDC-III	16 (7.4)	2 (10.0)
CDCA	12 (5.6)	0
LP	6 (2.8)	0
LACDC	4 (1.9)	1 (5.0)
Other	22 (10.2)b	1 (5.0)c
Behavioral health setting		
Private practice	57 (26.5)	11 (55.0)
Community-based outpatient treatment clinic	55 (25.6)	3 (15.0)
Hospital-based outpatient services	26 (12.1)	2 (10.0)
Prison/jail	12 (5.6)	2 (10.0)
School-based behavioral health	11 (5.1)	0
Hospital-based inpatient services	9 (4.2)	0
Child welfare agency	9 (4.2)	0
Residential treatment facility	8 (3.7)	0
Senior services	5 (2.3)	1 (5.0)
Managed care organization	4 (1.9)	0
Developmental disability services	4 (1.9)	0
Public health agency	3 (1.4)	0
Domestic violence agency	2 (0.9)	1 (5.0)
Other^d^	10 (4.7)	0
Years worked as a behavioral health provider (M, SD)	14.13 (10.20)	9.88 (6.23)
Years worked at the current organization (M, SD)	7.09 (7.57)	3.72 (4.09)

^a^Other race, chose not to disclose or preferred not to answer

^b^Other licenses included licensed marriage and family therapist, licensed independent marriage and family therapist, licensed alcohol and drug counselor, Certified Addiction Counselor-III, National Certified Addiction Counselor-II, substance use disorder professional, Certified Independent Professional, Certified Addiction Specialist, credentialed alcoholism and substance abuse counselor, person-centered case manager, licensed mental health counselor, licensed clinical addiction specialist, licensed addiction specialist, Certified Brain Injury Specialist, master addiction counselor, licensed school counselor, registered nurse, certified community health worker

^c^Other license was substance use disorder professional

^d^Other settings included primary care, military-based treatment setting, homeless shelter, community outreach and crisis center, affordable housing agency, employee assistance program, domestic violence shelter, local government authority, university academic medical institute, and professional ice hockey organization

**Fig. 2:**
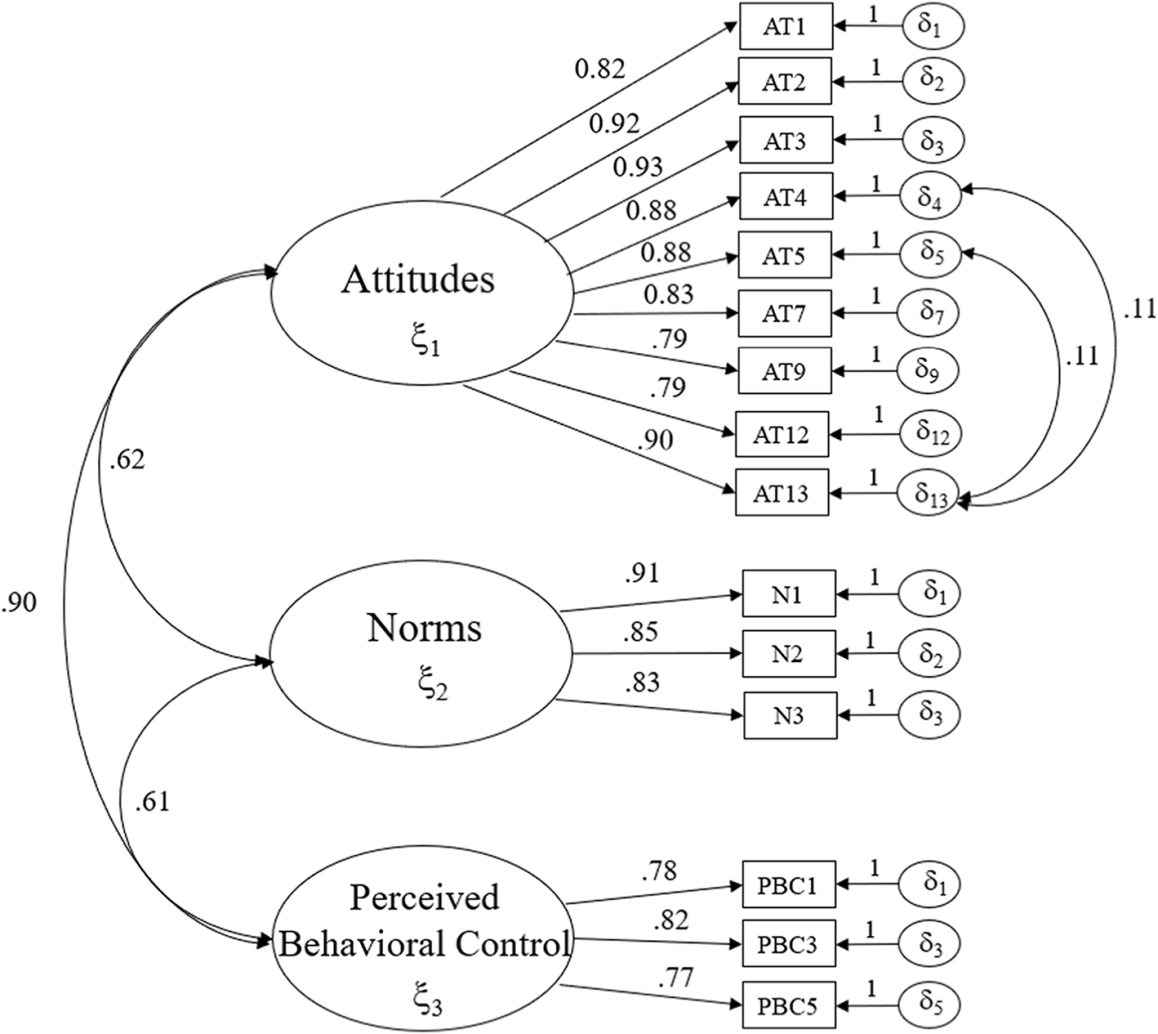
Fit of the measurement model with standardized estimates. Note: All factor loadings are significant at the *p* < .001 level

The measurement model yielded excellent fit incidences (*χ*^2^ = 303.63, *p* < 0.01; *CFI* = 0.98; *TLI* = 0.98; *SRMR* = 0.04; *RMSEA* = 0.11; 90% *CI* = 0.10–0.12). Because the upper bound of the 90% confidence interval for the RMSEA exceeded 0.06, residual correlations were confirmed to be equal to or less than one [12]. All the factor loadings of the measurement model presented in Fig. 2 are statistically significant (*p* < .001). In the general SEM model, the substantive path from PBC leading to intentions was not statistically significant (*p* = 0.09) and therefore removed. The final model yielded excellent model fit (*χ*^2^ = 346.13, *p* < 0.01; *CFI* = 0.98; *TLI* = 0.98; *SRMR* = 0.04; *RMSEA* = 0.09; 90% *CI* = 0.08–0.10), and all remaining paths were retained in the final model.

**Fig. 3:**
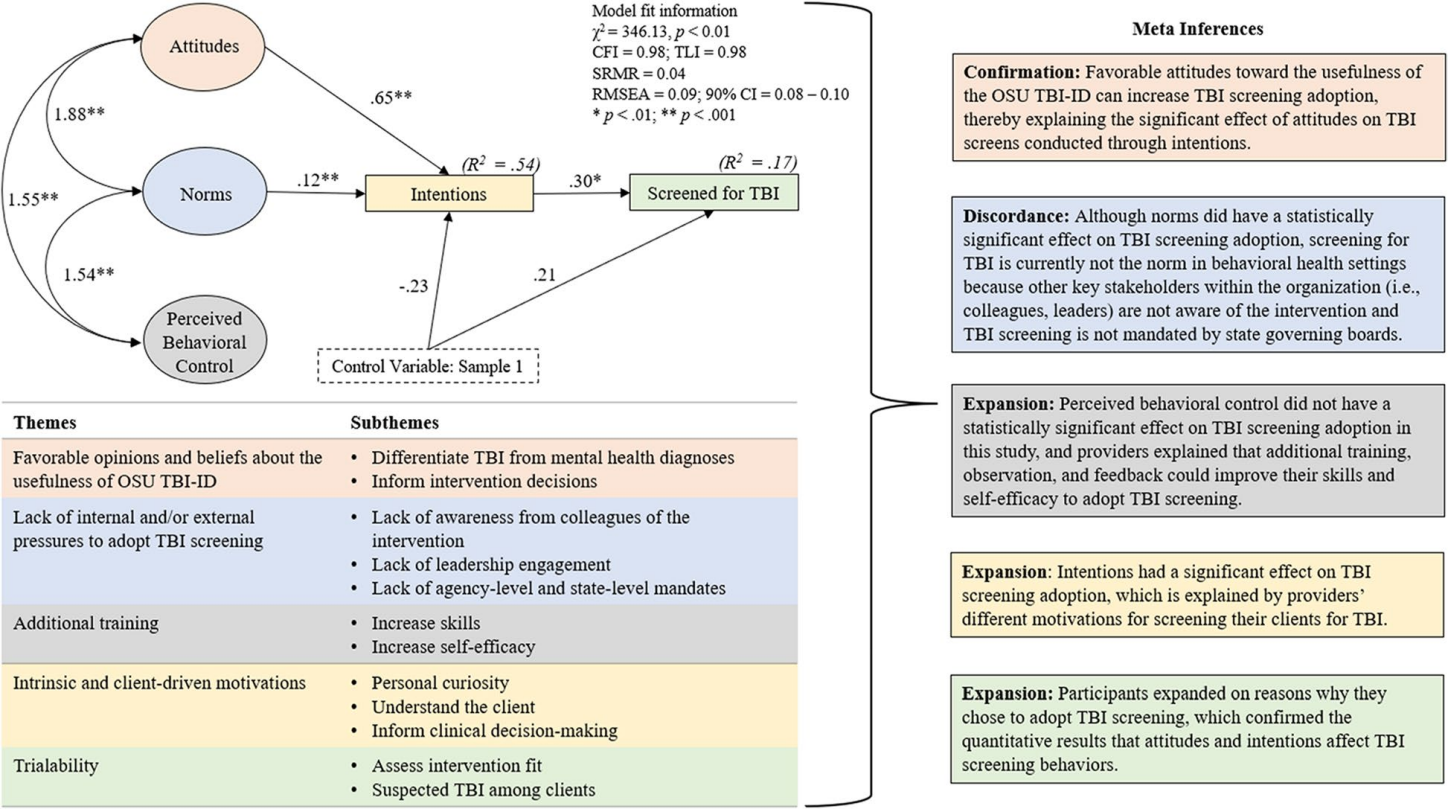
Joint display connecting the structural equation model results to the qualitative themes with meta-inferences

The following section presents the results from the SEM and the qualitative interviews woven together in the text on a construct-by-construct basis aligned with the TPB [58]. Figure 3 presents the unstandardized model results for the final SEM along with the main themes and subthemes from the qualitative results and meta-inferences. Table 5 is a joint display of the main themes and subthemes and direct participant quotes presented alongside the mean scores and standard deviations from the TPBQ-TBI subscales.

**Table 5:** Joint display of the quantitative and qualitative results connected to constructs from the TBP

TBP constructs	Themes	Subthemes	Sample quotes
TBPQ-TBI Mean and SD			
*Attitudes* 5.57 (0.92)	Favorable opinions and beliefs about the usefulness of OSU TBI-ID	• Differentiate TBI from mental health diagnoses • Inform intervention decisions	“I'm very positive about it. I certainly see the usefulness and it's great to be able to have guidelines to follow because I really do think that TBI is underreported and a huge part of some of the stuff that we could be doing better like identifying things like that. So having an actual screening tool is really helpful and kinda takes the guesswork out.” (Licensed Independent Social Worker with Supervision Distinction, Mitigation Specialist, prison setting) *“*You're able to discern or differentiate between somebody's psychiatric issues. Somebody's relapse warning. I mean it adds a whole other layer, somebody's overall whole, the whole person approach and its primary factor on substance use issues. We're talking about mental health issues and substance use disorders. Traumatic brain injuries [are] another primary medical concern that is of significance when trying to treat somebody” (Licensed Professional Clinical Counselor, Director of Outpatient Services, community-based treatment setting) “I think it would be beneficial to give us more information and maybe help guide some of the diagnoses or assessments with clients. So, I definitely think it would be useful.” (Licensed Professional Counselor, Therapist, community-based outpatient treatment setting)
Subjective norms 2.99 (0.92)	Lack of internal and/or external pressures to adopt TBI screening	• Lack of awareness from colleagues of the intervention • Lack of leadership engagement • Lack of agency-level and state-level mandates	“I mean those [colleagues] who are [screening for TBI], I can think of one in my office who is aware of the effects of TBI. She does [screen for TBI], she is likely asking those questions. I don't know that anybody else is.” (Licensed Professional Counselor, Therapist, community-based outpatient treatment setting) “If more of my colleagues were on board with it, to promote it in terms of it being useful to them as well, especially outside of integrated behavioral health…I wonder if the other providers would see the importance of it. Because, if you're in this field already of integrated behavioral health, you can see that it would be worth happening. But if you're not inside this field they may raise questions; they want to know why do you want it or why do you need it.” (Licensed Chemical Dependency Counselor-II, Addiction Counselor, community-based outpatient treatment setting) “I mean, to me it sounds like a very valuable service and everything, that in terms of the likelihood of it being incorporated and utilized, that would be an upper management decision and call. I feel that it would be very beneficial, but I don't have any input or access to voice my opinion and so I don't know what actually aids the implementation of the program.” (Licensed Social Worker, Therapist, community-based outpatient treatment setting)
Perceived behavioral control 4.42 (1.17)	Additional training needed to enhance skills and confidence	-	“I think I would need a lot more training, and hands on learning observation, and supervision to learn and implement the system before I feel comfortable jumping in on mine. I don't feel that I'm sufficiently trained at this time to do it on my own” (Licensed Social Worker Therapist, community-based outpatient treatment setting)
			“I don't think there are any expectations other than what I present to my leadership as this should be an expectation. So, I don't really have any standards to abide by, I kind of make it up as I go along. But I do believe that they would trust me enough to know that they should look into this … I know that they would be back.” (Licensed Independent Social Worker with Supervision Distinction, Mitigation Specialist, prison setting)
Intentions 3.34 (1.51)	Intrinsic- and client-driven motivations	• Personal curiosity • Understand the client • Inform clinical decision-making	“I kind of come from a place of curiosity and wanting to help versus here's another screening you have to do, this is just part of our intake, just fill it out, there's a reason, there's a purpose for it… It's just I want to make sure that I'm treating them in the best way possible with the most tools that are gonna be the most effective for them. And if they've had a traumatic brain injury, that really is hampering their quality of life. I am not qualified to treat that, I am qualified to refer and really encourage them to get that additional assessment and potentially treatment.” (Licensed Professional Counselor, Director, private practice) “It does give me a lot of guidance on if I need to ask, what I liked about it is that it gives me further guidance on what kind of questions I need to ask, where we're gonna look for referrals in order to get the client what they need.” (Licensed Social Worker, Crisis Counselor, Domestic Violence Shelter)
TBI screening adoption 25.6% of the total sample	Trialability	• To assess intervention fit • Suspected TBI among clients	“I just I ran through it and then I actually end up having a program participant in my chair and so I've got to ask him the questions. He doesn't have a TBI. He has no history of that at all. But I was just kinda like, I don't want to practice, but it was just I had just gotten the thing and I was like, ‘Let's see how this works’. And so, you know, I sat down with him and pretended.” (Licensed Professional Chemical Dependency Counselor-II, Correctional Program Coordinator, prison setting)
			“One of them is a death penalty client, and so I really wanted to cover all the mitigation bases because I feel like that it's life or death, right? Might as well do everything I certainly have available to me to either look into things or rule out things. And the other guy I'd been lost. I'd been absolutely lost on where to go with him … to help me understand how I can best help him even in my mitigation, like okay, you're 30 years old and then you snapped. Well, that could have a lot to do with your mental health, but are there other things going on?” (Licensed Independent Social Worker with Supervision Distinction, Mitigation Specialist, prison setting) “Well, I mean, because I had done the training and I was like, I really wanted to see how successful I could make it in the assessment process and so it was really just kind of a trial for me, and I don't normally do assessments, like I supervise people who did their assessment, so I don't normally act myself. So I had to kind of like, I took this client, I'm gonna do this assessment, and this time I'm gonna incorporate it. So it was really a trial to see how easy it would be to incorporate it in, that's why I did that.” (Licensed Independent Social Worker with Supervision Distinction, Assessment Supervisor, senior services)

### Attitudes

TPBQ-TBI subscale scores demonstrated favorable attitudes toward using the OSU TBI-ID to screen for TBI (*M* = 5.57, *SD* = 0.92) (Table 5). In SEM, intentions to screen for TBI using the OSU TBI-ID fully mediated the relationship between attitudes and TBI screening behaviors. Specifically, providers who reported more favorable attitudes at the Time 1 assessment demonstrated increased odds of screening for TBI at the Time 2 assessment (*OR* = 0.65, *SE* = 0.09, *p* < .001).

This finding was confirmed by the qualitative interviews, where interview participants reported favorable opinions toward and beliefs about the usefulness of the OSU TBI-ID. Specifically, providers reported that screening for TBI using this method would help them to differentiate mental health or substance use disorders from a TBI by gaining additional insight into the client’s clinical presentation and problems presented during the assessment. Providers reported that knowing a client has a history of TBI could offer greater insight into differential diagnoses or possible sources of post-traumatic stress disorder (PTSD), attention-deficit hyperactivity disorder (ADHD), or identify changes to mood potentially due to the TBI. A provider explained the following:

With trauma and with ADHD, processing can be affected by brain injury. So, knowing that may be a cause or part of what's going on, as far as mental health diagnoses, would be beneficial… [TBI] might mimic another diagnosis, so that's really powerful. [Licensed Professional Counselor, Therapist, community-based outpatient treatment setting]

Providers also explained that because of the utility of this screening method to differentiate possible symptoms of TBI from mental health or substance use disorders, their intervention decisions could be better directed. Specifically, providers explained that their treatment plans and/or referrals could be better tailored to the individual client.

### Subjective norms

The mean score for subjective norms was 2.99 (*SD* = 0.92). In SEM, intentions to screen for TBI using the OSU TBI-ID fully mediated the relationship between subjective norms and TBI screening behaviors. Specifically, providers who reported higher subjective norms at Time 1 demonstrated increased odds of screening for TBI at Time 2 (*OR* = 0.12, *SE* = 0.06, *p* < .01). These quantitative results were discordant with the qualitative interviews, where the main theme was an overall lack of internal and/or external pressures to adopt TBI screening; however, this was highly context dependent. In private practice settings, providers discussed limited pressures to adopt new screening methods. A provider explained the following:

I definitely don't [screen for TBI] because I'm an independent contractor and definitely I guess it would be up to my own judgement… I’m in a private practice setting, so there would be other clinicians, but we all operate independently. So, it's a matter of like … everyone does their own assessments in their own practice. [Licensed Independent Social Worker, Therapist, private practice setting]

In group-based practice settings, however, colleagues were generally unaware of TBI and its implications on practice and, subsequently, did not pressure each other to adopt this screening method. Specifically, providers reported that if other colleagues were also using the OSU TBI-ID, then they might be more willing to adopt it. In addition, subjective norms were affected by the lack of leadership engagement needed to nudge providers to adopt TBI screening, as well as the lack of organizational-level and state-level mandates that would require TBI screening to be adopted. A provider explained the following:

Where I see an issue, and I think this is an issue with any type of change or any type of new program that comes in, is that it's not mandated. Staff has a really hard time incorporating something that is outside of what their mandate is for …. Anything that comes down as ‘we must do this’ is based on a funder … [Licensed Independent Social Worker with Supervision Distinction, Assessment Supervisor, senior services setting]

Although many participants explained that they have taken steps to discuss with their organization’s leader about this screening method, overall, leaders have not yet initiated actions to increase widespread adoption, such as offering training or continued education opportunities on TBI for providers employed within the organization.

### Perceived behavioral control

The mean score on the “PBC” subscale was 4.42 (*SD* = 1.17). In SEM, neither the direct path from PBC on TBI screening behaviors nor the indirect path through intentions on behaviors were significant. Interview participants, however, expanded on PBC by explaining that once they had a chance to practice implementing TBI screening, then their confidence also increased. However, the main theme was that providers described the desire to obtain additional training, education, and direct observation to enhance their skills and confidence needed to adopt the OSU TBI-ID. Interview participants discussed that although learning the screening method itself was relatively simple, more education on how TBI relates to behavioral health, as well as what to do following a positive TBI screen, are necessary before they would feel comfortable enough with TBI screening. These qualitative results help to explain why the quantitative measure of PBC may not have had a direct or indirect effect on TBI screening adoption in SEM.

### Intentions

The mean score on the “Intention” subscale was 3.34 (*SD* = 1.51). In SEM, providers who reported greater intentions to screen for TBI at Time 1 demonstrated greater odds of adopting the OSU TBI-ID at Time 2 (*OR* = 0.30, *SE* = 0.10, *p* < .001). Attitudes and subjective norms accounted for 54% of the variance in intentions (*R*^*2*^ = 0.54).

Qualitative interview results expanded upon these quantitative results. Specifically, intentions to screen for TBI were based on intrinsic and client-driven motivations. Regarding intrinsic motivations, some interview participants reported simply wanting to practice conducting the intervention. These participants explained that they were curious about how this method worked in practice with clients, which drove their motivation to conduct the screening. Other participants explained that they had experienced a TBI themselves, which drove their intentions to use this screening method to identify TBI among clients. Interview participants also explained that a primary motivation to conduct TBI screening stemmed from wanting to make more informed referrals or treatment plans or to better understand a client with complex symptoms.

### TBI screening adoption

Only 25% (55/215) of the sample reported having screened for TBI during the 1-month period (range: 1–40, *M* = 4.49, *Mdn* = 2.0, *SD* = 6.27). Providers with masters or doctoral degrees were more likely to adopt TBI screening compared to providers with associate or bachelor’s degrees (89.1% versus 10.9%, respectively, *p* = 0.02). In addition, providers employed in private practices reported being significantly more likely to adopt TBI screening compared with providers employed in non-private practice settings (*p* < 0.01; *Φ* = 0.33). In SEM, intentions accounted for 17% of the variance in TBI screening behaviors (*R*^*2*^ = 0.17).

Providers who participated in the qualitative interviews expanded on the primary reason why they chose to adopt TBI screening, which was trialability. Specifically, providers who adopted the OSU TBI-ID chose to do so to assess intervention fit within their current workflows and specifically into biopsychosocial assessments. Providers who adopted the screening method also explained that because they suspected TBI among their clients, they wanted to trial the intervention to confirm their beliefs about the presence of TBI among clients. One provider discussed the importance of using the screening intervention in her work with survivors of domestic violence:

I work for [a domestic violence shelter], and for me personally, I think that it's very important. I actually approached my clinical director with this screening tool and the education part of things because our statistics do show, and from just the evidence of working with our clients, that 83% of our individuals that have experienced intimate partner violence do have at least one TBI. A lot of them are going unaddressed. It's very important, and for me that's extremely important as the crisis clinician here to be able to know whether that's something that we might be working with them….” [Licensed Social Worker, Crisis Counselor, Domestic Violence Shelter]

## Discussion

The current study investigated how characteristics of behavioral health providers (i.e., attitudes, PBC, subjective norms) affect the adoption of the OSU TBI-ID in behavioral healthcare settings and contextualized these results through qualitative interviews. We found that more favorable attitudes and greater norms were associated with increased odds of intentions to screen for TBI and TBI screening behaviors; however, PBC did not have a significant effect on intentions or behaviors in this study. Results from the qualitative interviews demonstrated that behaviors were driven by providers’ motivations to trial the OSU TBI-ID to assess for intervention fit within their current workflows or to determine exposure to TBI among clients.

This study is the first to examine the early adoption of TBI screening in behavioral healthcare contexts. Our theory-driven, mixed-methods approach allows for greater contextual understanding about how provider-level characteristics and other multilevel contextual factors affect adoption of the OSU TBI-ID in behavioral healthcare settings in the USA while also offering unique contributions to the implementation science literature. Specifically, we grounded our study in the TPB to specify causal relationships between constructs [65] necessary to building predictive models. In the early stages of implementation, the TPB is particularly advantageous to understanding how characteristics of providers affect TBI screening adoption [23]. Second, our mixed-methods approach provides a deeper and more nuanced explanation about *how* and *why* provider-level characteristics affect TBI screening adoption observed in this study by contextualizing quantitative results with the addition of the qualitative component [64, 66]. Our qualitative results illuminated several deeper explanations about why some providers chose to adopt screening while others did not and revealed additional multilevel determinants affecting TBI screening adoption in behavioral healthcare. These additional determinants provide a theoretical base for building future models that situate these constructs as mediators, moderators, and mechanisms to test in future studies [67]. Strategies can therefore be selected beyond those that map back to individuals and extend to outer- and inner-setting factors to stage implementation scale-up.

Our results demonstrated that providers’ attitudes toward using the OSU TBI-ID to screen for TBI were generally positive, and that these attitudes had an indirect effect on TBI screening behaviors through intentions, which is consistent with other literature guided by the TPB [27, 68, 69]. Qualitative interviews revealed that providers’ attitudes were shaped by their perceptions about the usefulness of the OSU TBI-ID in facilitating assessments by better identifying and delineating mental health problems from TBI. Furthermore, providers’ perceptions about the usefulness of TBI screening were shaped by their beliefs about using the results of the OSU TBI-ID to guide clinical decision-making by either driving referrals to specialized services or by guiding current treatment approaches that take into account, for instance, the clients’ memory problems or ability to process information. These results have implications for the role of provider attitudes as proximal mechanisms in the progression toward behavior change [70, 71]. Although attitudes do not directly impact behaviors, they do serve as proximal change mechanisms leading to adoption, and hence, understanding provider attitudes toward an innovation in the pre-implementation stages can lead to more a precise selection of implementation strategies and how these strategies should be tailored based on baseline attitudes. In this pre-implementation study, we utilized training as a means to raising awareness about the OSU TBI-ID so that we could assess attitudes toward this innovation. Although training alone is insufficient to increase adoption of TBI screening [72, 73], training is still a necessary first step needed to inform attitudes and lay the foundation for building provider-level capacity to screen for and treat clients with co-occurring TBI and behavioral health conditions. However, additional implementation strategies beyond training will be necessary for increasing TBI screening uptake. Specifically, bundling training with consultation and/or tailored educational efforts to specific provider groups about the OSU TBI-ID and its usefulness in clinical practice could have benefits in improving initial attitudes toward TBI screening that ultimately lead to increased adoption [71, 72]. However, identifying, testing, and specifying the mechanisms through which these implementation strategies operate on EBP adoption are necessary to reduce implementation costs and expedite the public health benefit of EBPs through more precise implementation approaches. Nonetheless, given that over half of clients in behavioral healthcare settings have a lifetime exposure to TBI that affects their ability to fully engage in and benefit from treatment [3, 74], tailored training and educational strategies are important first steps to changing the treatment landscape.

Consistent with TPB and other implementation studies guided by the TPB [26, 27, 68], our quantitative results demonstrated that social pressures drove intentions and screening behaviors. Specifically, we found that when providers perceived higher social pressures to screen for TBI, that TBI screening intentions and behaviors were also higher. However, our qualitative results suggested that social pressures to screen were relatively minimal across various types of behavioral health settings represented in this study. Overall, providers explained that minimal internal pressures exist to adopt TBI screening, which was primarily attributed to inadequate awareness from leadership and colleagues about TBI. Providers employed in community-based practice settings also reported minimal external pressures by state-level funders to adopt TBI screening. It is therefore possible that screening adoption in this study was low due to inadequate intraorganizational social pressures needed to nudge provider behavior change [27, 75], as well as the absence of state-level policies mandating or incentivizing TBI screening. Specifically, providers working in community-based organizations that rely on funding from grants and contracts from public agencies may be more exposed to external pressures than private practice providers. Therefore, different implementation strategies may be needed to target inner-setting or outer-setting determinants depending on the type of organization and their funding structures [29]. Specifically, for publicly funded organizations, buy-in from state-level leadership and/or mental health and substance use treatment boards may be a top-down approach necessary to shift social norms or to mandate the use of the OSU TBI-ID in these organizations. In private practice settings, implementation strategies that target provider motivations and build buy-in to conduct TBI screening, such as local consensus discussions and educational outreach visits [76], may be warranted. In both settings, policy-level funding structures that reimburse for time to conduct the screening could incentivize providers to screen their clients for brain injury. Beyond testing these strategies, more research is needed to better understand the mechanisms through which these multilevel strategies might influence social norms within each type of setting.

Similar to other implementation science literature guided by the TPB [30], we found that PBC did not demonstrate a significant effect on TBI screening adoption. Notably, contrary to other implementation studies [26, 27, 30], we contextualized our quantitative results through qualitative interviews with providers to understand why PBC did or did not have an effect on adoption. Providers explained that this lack of perceived control was due to insufficient knowledge, skills, or self-efficacy needed to adopt TBI screening. Consistent with prior research [77], most of the providers who participated in the qualitative interviews reported believing that TBI is a medical issue and were unable to articulate the connection between TBI, mental health, and substance use disorders, as well as their role in TBI identification. Furthermore, providers reported hesitation to adopt TBI screening due to inadequate knowledge about what steps to take following a positive TBI screen. Although most providers reported that identifying a TBI could guide clinical decision-making, providers did not know how to tailor treatment approaches or where to make referrals, which likely contributed to their beliefs about difficulty in conducting TBI screens. These results confirm prior research [77, 78] and point to the need for more comprehensive education on the connections between TBI, mental health, and substance use disorders, as well as strategies to support implementation uptake, such as facilitation.

Taken together, these individual-level determinants help to explain why only one-quarter of providers in this study adopted TBI screening during the study period while also pointing to other inner-setting and outer-setting factors affecting adoption. Notably, although the training modules we used for this study were not meant to have a significant impact on adoption of TBI screening at this stage in the implementation process since we were interested in understanding pre-implementation contextual factors, future implementation efforts should bundle active, ongoing training with other implementation strategies, such as consultation or implementation facilitation, to intentionally engage and support providers and leaders from pre-implementation through sustainment to ensure that TBI screening and treatment become embedded and normalized into behavioral health practice [71, 72, 79]. Implementation facilitation might be a particularly useful implementation strategy applied to TBI research-to-practice translation in behavioral health treatment contexts. Although facilitation has garnered a growing body of evidence in the support of adoption, implementation, fidelity, and maintenance of a variety of EBPs [80–83], more research is needed to understand if and how facilitation might be useful in integrating TBI screening and treatment into behavioral healthcare.

Interventions that address complex physical and mental health comorbidities into behavioral healthcare contexts remain understudied in the implementation science literature [32]. This study begins to address this gap by illuminating some of the determinants that affect service integration for complex conditions in behavioral healthcare. More specifically, this study is the first to investigate early determinants affecting the translation and implementation of the OSU TBI-ID into behavioral healthcare settings. Translating this screening method from research into practice is particularly challenging because TBI is often viewed as a medical condition to be identified and addressed by medical professionals. Although TBI does sometimes require intensive medical intervention to address the physical effects of the injury (i.e., neuroendocrine dysfunction or subdural hematoma, for example), vast evidence has demonstrated that TBI can result in chronic cognitive, behavioral, and psychiatric conditions over the life course [84, 85], which disproportionately affects individuals who seek care in behavioral health settings [3]. Despite how common TBI is in these settings, behavioral health providers often do not receive any formal education on TBI and hence enter the workforce unaware about the presence of TBI among clients or their roles in addressing these clients’ needs. Comprehensive education during undergraduate and graduate training programs that incorporate education on TBI into relevant curriculums is one strategy that begins to address this core issue. In addition, pointed efforts to train the existing workforce on how to administer the OSU TBI-ID and provide tailored treatment within the practice setting are also implementation strategies that begin to address provider knowledge, change beliefs, and improve confidence in adopting TBI screening and treatment [76, 78, 86–88]. However, even when formal education and training are provided, additional strategies will still be needed to address workforce capacity to treat these clients, particularly given the additional multilevel determinants at play that will inevitably hamper implementation efforts. Building upon prior implementation research and the results from this study, implementation strategies should be selected and tested for their effectiveness on improving EBP adoption, implementation, fidelity, sustainment, and scaling within the context of TBI research-to-practice translation. Multifaceted implementation strategies, such as facilitation [81], which address both provider- and inner-setting determinants like leadership engagement or implementation climate [80, 83], for example, are promising approaches to begin addressing this issue. Outer-setting implementation strategies that target policy-level change efforts will also be pivotal to creating system-level changes that can sustain these interventions long term. Specifically, involving state regulatory boards or state department leadership is top-down approaches that could stimulate change, as well as altering financial structures to reimburse TBI screening and treatment [89].

This study has several limitations. First, the heterogeneity of the sample may have limited our understanding about TBI screening adoption within specific types of behavioral health settings. Specifically, private practice settings inherently differ from domestic violence or community-based substance use treatment settings, for example, which may in turn affect the extent to which TBI screening is adopted overall. In addition, attitudes, norms, and PBC are likely to also differ between settings which may have been one reason why attitudes and norms had a significant effect on adoption, but PBC did not. Therefore, future research could investigate differences in determinants across types of behavioral health settings using a more granular approach. Second, although our sampling frame broadened the reach of our study to providers from various states, disciplines, and educational backgrounds, it may have also contributed to the variability in some of our results. Although significant differences between samples were controlled for in the advanced analysis, future studies should replicate these findings with a larger sample with sufficient subgroups of settings to allow for variance to be explored more thoroughly. Another limitation was voluntary response bias. It is possible that providers who elected to participate in this study already had an interest in TBI, potentially leaving out perspectives of providers without vested interest but who may be treating clients with TBI without knowing it. Similarly, the self-report nature of TBI screening behaviors could have resulted in under or overestimation of the actual number of TBI screens conducted. Future research should prospectively track the number of TBI screens conducted in real time to gain a more accurate picture of behaviors. A limitation also existed with regard to attrition bias between the two timepoints. It is possible that providers self-selected out of the study after Time 1 because they believed that TBI screening is not relevant to their clients or practice settings. Another possible limitation is desirability bias, which is a potential explanation for discrepancy between some of the quantitative and qualitative results. Finally, because no standard measure exists for the TPB [90], we had to adapt our TPBQ-TBI measure from prior literature [32]. Although our CFA determined a strong measurement model, future studies are needed to establish a common measure for research applying this theory.

## Conclusions

This is the first study to investigate implementation of TBI screening in behavioral healthcare settings, which represents a critical shift in the way in which traditional TBI research has been conducted. Specifically, our study is the first step in the translation of EBPs for individuals with co-occurring TBI, mental health, and substance use disorders into behavioral healthcare settings, which closes a critical research-to-practice gap using implementation science. Our work also represents the first step in advancing an overall implementation science research agenda by specifying and testing theory-driven constructs as predictors and mediators on EBP adoption [91] and serves as a basis to identifying implementation strategies that span characteristics of individuals and outer-setting and inner-setting domains to tailor and test in future research.

### Supplementary Information

**Additional file 1: Supplemental file 1.** Mixed Methods Article Reporting Standards: Information on the Collection and Integration of Qualitative and Quantitative Data [19].

**Additional file 2: Supplemental file 2.** Sample characteristics of participants from Phase I.

**Additional file 3: Supplemental file 3.** Descriptive Statistics of the Constructs from the Theory of Planned Behavior by Sub-Sample.

## Data Availability

De-identified data are available upon reasonable request by contacting the lead author, Kathryn A. Hyzak.

## Abbreviations

CFI: Comparative fit index
EBPs: Evidenced-based practices
FIML: Full information maximum likelihood
JARS-MMR: Journal Article Reporting Standards for Mixed-Methods Research
MCAR: Little’s test of missing completely at random
MVA: Missing values analysis
OSU TBI-ID: Ohio State University Traumatic Brain Injury Identification Method
RMSEA: Root-mean-square error of approximation
SRMR: Standardized root-mean-square residual
SEM: Structural equation modeling
TBI: Traumatic brain injury
TPB: Theory of Planned Behavior
TPBQ-TBI: Theory of Planned Behavior Questionnaire for TBI
TLI: Tucker-Lewis index
US: United States
WLSMV: Weighted least-squares mean and variance
