# Supplementary material for: Implementing traumatic brain injury screening in behavioral health treatment settings: results of an explanatory sequential mixed-methods investigation
Source: Implement Sci. 2023 Aug 16;18:35. doi: 10.1186/s13012-023-01289-w (PMC10428542; doi:10.1186/s13012-023-01289-w)
Supplement: Supplementary file 2 — Additional file 2: Supplemental file 2. Sample characteristics of participants from Phase I. [file 13012_2023_1289_MOESM2_ESM.docx]

| Table 1.  Supplemental File 2.  *Sample characteristics* *of participants from Phase I* | | | | | | | |  |
| --- | --- | --- | --- | --- | --- | --- | --- | --- |
|  | Total  (N = 215) | Sample 1  (n = 15) | Sample 2  (n = 23) | Sample 3  (n = 130) | Sample 4  (n = 47) | *Range* | *p*^a^ | Effect size^b^ |
|  | N | n (%) | n (%) | n (%) | n (%) |  |  |  |
| Age Group |  |  |  |  |  |  | 0.18 | 0.28 |
| 18 – 24 | 4 (1.9) | 0 (0.0) | 0 (0.0) | 4 (3.1) | 0 (0.0) |  |  |  |
| 25 – 34 | 45 (21.2) | 2 (13.3) | 4 (18.2) | 33 (25.6) | 6 (13.0) |  |  |  |
| 35 – 54 | 97 (45.8) | 6 (40.0) | 15 (68.2) | 57 (44.2) | 19 (41.3) |  |  |  |
| 55 – 65 | 49 (23.1) | 5 (33.3) | 3 (13.6) | 25 (19.4) | 16 (34.8) |  |  |  |
| > 65 | 17 (8.0) | 2 (13.3) | 0 (0.0) | 10 (7.8) | 5 (10.9) |  |  |  |
| Gender |  |  |  |  |  |  | 0.89 | 0.10 |
| Female | 181 (85.4) | 13 (86.7) | 20 (90.9) | 111 (86.0) | 37 (80.4) |  |  |  |
| Male | 30 (14.2) | 2 (13.3) | 2 (9.1) | 17 (13.2) | 9 (19.6) |  |  |  |
| Nonbinary | 1 (0.5) | 0 (0.0) | 0 (0.0) | 1 (0.8) | 0 (0.0) |  |  |  |
| Race/Ethnicity |  |  |  |  |  |  | 0.02* | 0.37^c^ |
| Caucasian or White | 176 (81.9) | 12 (80.0) | 18 (78.3) | 114 (87.7)** | 32 (68.1)** |  |  |  |
| African American or Black | 16 (7.4) | 3 (20.0) | 1 (4.3) | 8 (6.2) | 4 (8.5) |  |  |  |
| Multi-Racial | 11 (5.1) | 0 (0.0) | 1 (4.3) | 4 (3.1) | 6 (12.8) |  |  |  |
| Hispanic or Latinx | 6 (2.8) | 0 (0.0) | 1 (4.3) | 1 (0.8)** | 4 (8.5)** |  |  |  |
| Asian or Pacific Islander | 2 (0.9) | 0 (0.0) | 0 (0.0) | 2 (1.5) | 0 (0.0) |  |  |  |
| Other^c^ | 4 (1.8) | 0 (0.0) | 2 (9.5) | 1 (0.8) | 1 (2.2) |  |  |  |
| Highest Level of Education |  |  |  |  |  |  | 0.09 | 0.17 |
| Masters or Doctorate | 166 (78.3) | 14 (93.3) | 21 (91.3) | 99 (77.3) | 32 (69.6) |  |  |  |
| Associates or Bachelors | 46 (21.7) | 1 (6.7) | 2 (8.7) | 29 (22.7) | 14 (30.4) |  |  |  |
| License Type |  |  |  |  |  |  |  |  |
| LSW | 58 (27.0) | 4 (2.7) | 3 (13.6) | 48 (36.9)** | 3 (6.4)** |  | < .01 | 0.30 |
| LISW-S | 47 (21.9) | 4 (2.7) | 2 (9.5) | 35 (26.9) | 6 (12.8) |  | 0.08 | 0.18 |
| LPC | 24 (11.2) | 1 (6.7) | 7 (30.4)** | 4 (3.1)** | 12 (25.5) |  | < .01 | 0.36 |
| LISW or LCSW | 23 (10.7) | 3 (20.0) | 2 (9.5) | 15 (11.5) | 3 (6.4) |  | 0.48 | 0.12 |
| LICDC | 22 (10.2) | 2 (13.3) | 1 (4.3) | 14 (10.8) | 5 (10.6) |  | 0.78 | 0.07 |
| LPCC or LPCC-S | 19 (8.8) | 1 (6.7) | 4 (21.1) | 13 (10.0) | 1 (2.1) |  | 0.17 | 0.15 |
| LCDC-II or LCDC-III | 16 (7.4) | 1 (6.7) | 2 (9.5) | 3 (2.3)** | 10 (21.2)** |  | < .01 | 0.29 |
| CDCA | 12 (5.6) | 1 (6.7) | 0 (0.0) | 6 (4.6) | 5 (10.6) |  | 0.27 | 0.14 |
| LP | 6 (2.8) | 1 (6.7) | 4 (18.2)** | 0 (0.0)** | 1 (2.1) |  | <.01 | 0.33 |
| LACDC | 4 (1.9) | 0 (0.0) | 0 (0.0) | 0 (0.0) | 4 (8.5) |  | <.01 | 0.26 |
| Other | 27 (12.6) | 1 (6.7) | 0 (0.0) | 5 (3.8)** | 15 (31.9)** |  | <.01 | 0.42 |
| Behavioral Health Setting |  |  |  |  |  |  | 0.02* | 0.51 |
| Private practice | 57 (26.5) | 7 (46.7)** | 14 (60.9) | 22 (16.9)** | 14 (29.8) |  |  |  |
| Community-based outpatient treatment clinic | 55 (25.6) | 2 (13.3) | 4 (17.4) | 36 (27.7) | 13 (27.7) |  |  |  |
| Hospital-based outpatient services | 26 (12.1) | 3 (20.0) | 2 (8.7) | 14 (10.8) | 7 (14.9) |  |  |  |
| Prison/jail | 12 (5.6) | 1 (6.7) | 0 (0.0) | 9 (6.9) | 2 (4.3) |  |  |  |
| School-based behavioral health | 11 (5.1) | 0 (0.0) | 0 (0.0) | 11 (8.5) | 0 (0.0) |  |  |  |
| Hospital-based inpatient services | 9 (4.2) | 0 (0.0) | 0 (0.0) | 8 (6.2) | 1 (2.1) |  |  |  |
| Child welfare agency | 9 (4.2) | 0 (0.0) | 0 (0.0) | 9 (6.9) | 0 (0.0) |  |  |  |
| Residential treatment facility | 8 (3.7) | 0 (0.0) | 1 (4.3) | 3 (2.3) | 4 (8.5) |  |  |  |
| Senior services | 5 (2.3) | 1 (6.7) | 0 (0.0) | 4 (3.1) | 0 (0.0) |  |  |  |
| Managed care organization | 4 (1.9) | 0 (0.0) | 0 (0.0) | 3 (2.3) | 1 (2.1) |  |  |  |
| Developmental disability services | 4 (1.9) | 0 (0.0) | 0 (0.0) | 4 (3.1) | 0 (0.0) |  |  |  |
| Public health agency | 3 (1.4) | 0 (0.0) | 0 (0.0) | 3 (2.3) | 0 (0.0) |  |  |  |
| Other^d^ | 12 (5.6) | 1 (6.7) | 2 (8.7) | 4 (3.1) | 5 (10.6) |  |  |  |
| Years worked as a behavioral health provider (M, SD) | 14.13 (10.20) | 14.93 (11.15) | 12.80 (7.41) | 14.21 (11.07) | 14.30 (8.66) | < 1 – 45 | 0.92 | 0.002 |
| Years worked at the current organization (M, SD) | 7.09 (7.57) | 7.43 (9.46) | 6.24 (4.89) | 7.40 (8.07) | 6.53 (6.61) | < 1 – 40 | 0.85 | 0.004 |
| ^a^ *P*-values are based on χ^2^ tests or Fisher’s exact test for categorical variables or One-way ANOVA for continuous variables.  ^b^ Effect sizes are based on Phi values for categorical variables or eta-squared for continuous variables (small = 0.1 < 0.3; medium = 0.3 < 0.5; large ≥ 0.5)  ^c^ Other Race = chose not to disclose or preferred not to answer.  ^d^ Other organizations include primary care, military-based treatment setting, homeless shelter, community outreach and crisis center, affordable housing agency, employee assistance program, domestic violence shelter, local government authority, university academic medical institute, and professional ice hockey organization.  * Significant at the *p* < .05 level  ** Post-hoc analyses demonstrated significant differences at the *p* < .05 level | | | | | | | |  |
