# Supplementary material for: Implementing traumatic brain injury screening in behavioral health treatment settings: results of an explanatory sequential mixed-methods investigation
Source: Implement Sci. 2023 Aug 16;18:35. doi: 10.1186/s13012-023-01289-w (PMC10428542; doi:10.1186/s13012-023-01289-w)
Supplement: Supplementary file 3 — Additional file 3: Supplemental file 3. Descriptive Statistics of the Constructs from the Theory of Planned Behavior by Sub-Sample. [file 13012_2023_1289_MOESM3_ESM.docx]

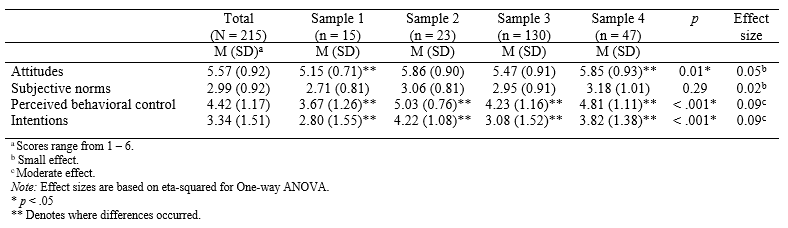
Supplemental File 3. *Descriptive Statistics of the Constructs from the Theory of Planned Behavior by Sub-Sample*
